# Logic modeling and the ridiculome under the rug

**DOI:** 10.1186/1741-7007-10-92

**Published:** 2012-11-21

**Authors:** Michael L Blinov, Ion I Moraru

**Affiliations:** 1 Center for Cell Analysis and Modeling, University of Connecticut Health Center, Cell and Genome Sciences Building, 400 Farmington Ave, Farmington, CT 06030-6406, USA

## Abstract

Logic-derived modeling has been used to map biological networks and to study arbitrary functional interactions, and fine-grained kinetic modeling can accurately predict the detailed behavior of well-characterized molecular systems; at present, however, neither approach comes close to unraveling the full complexity of a cell. The current data revolution offers significant promises and challenges to both approaches - and could bring them together as it has spurred the development of new methods and tools that may help to bridge the many gaps between data, models, and mechanistic understanding.

Have you used logic modeling in your research? It would not be surprising if many biologists would answer no to this hypothetical question. And it would not be true. In high school biology we already became familiar with cartoon diagrams that illustrate basic mechanisms of the molecular machinery operating inside cells. These are nothing else but simple logic models. If receptor and ligand are present, then receptor-ligand complexes form; if a receptor-ligand complex exists, then an enzyme gets activated; if the enzyme is active, then a second messenger is being produced; and so on. Such chains of causality are the essence of logic models (Figure 1a). Arbitrary events and mechanisms are abstracted; relationships are simplified and usually involve just two possible conditions and three possible consequences. The presence or absence of one or more molecule, activity, or function, [some icons in the cartoon] will determine whether another one of them will be produced (created, up-regulated, stimulated) [a 'positive' link] or destroyed (degraded, down-regulated, inhibited) [a 'negative' link], or be unaffected [there is no link]. The icons and links often do not follow a standardized format, but when we look at such a cartoon diagram, we believe that we 'understand' how the system works. Because our brain is easily able to process these relationships, these diagrams allow us to answer two fundamental types of questions related to the system: why (are certain things happening)? What if (we make some changes)?

## Untangling the ridiculome

**Figure 1:**
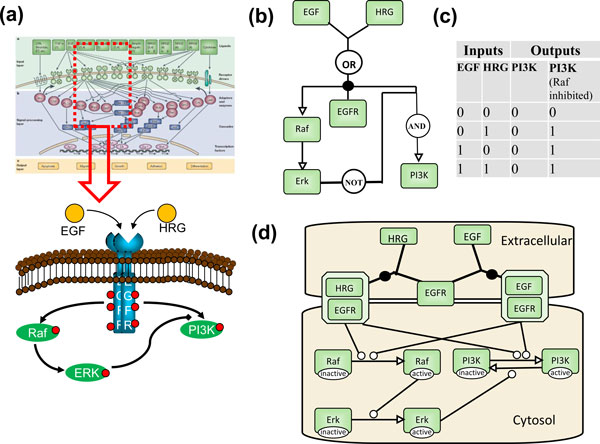
**Model representations**. **(a) **Typical cartoon diagrams and schematic interactions (adapted from [28]). Shapes, styles and colors are arbitrary. **(b) **The zoomed-in part of the cartoon diagram in (a) translated into a logic model. Shapes and arrow styles are represented in Systems Biology Graphical Notation (SBGN) standard (Entity Relationship (ER) format) that provide a defined one-to-one correspondence with a logic formalism. Arrows correspond to activation reactions. **(c) **A truth table corresponding to the logic model in (b) that shows how presence (1) or absence (0) of molecules of the input nodes leads to presence or absence of activity in the selected molecules of interest (output nodes). **(d) **Dynamic representation of the same model in SBGN standard (Process Diagram (PD) format). It can be considered either as a logic model or as a reaction diagram for a kinetic time-course simulation, where every node represents concentration of a chemical species.

But how about looking at a similar diagram that contains thousands of components, interconnected near and far? We may be able to infer the properties of certain subsystems, but we would not intuitively be able to predict overall behavior; to understand it as a whole. This is exactly what led to the development of formal logic-based modeling applications in biology. Even somebody with little mathematics training can recognize that the causal relationships represented in Figure 1a could be encoded by the states of the individual components (the system variables) described in their simplest form as absent (logical value FALSE, or 0) or present (logical value TRUE, or 1), and connected by the logical operators AND, OR, and NOT. Formally, this representation is called a Boolean network, and is typically represented as a graph (Figure 1b). Changes in the variable values can be computed by trivial Boolean algebra, and for small systems they are typically presented as 'truth tables', which show relationships between selected inputs and outputs (Figure 1c). This is a mathematical formalism that also happens to be the foundation upon which the entire digital world has been built, ever since Claude Shannon showed more than half a century ago how to use simple analog switches to perform binary computations. A human may feel helpless when facing a huge reaction network, but a computer can simulate the corresponding Boolean network in a fraction of a second; the state of the network under varying conditions and subject to arbitrary perturbations can be predicted and analyzed almost effortlessly. The algorithms and software implementations for this were perfected decades ago, and are routinely used by engineers to correctly simulate the behavior of circuits with enormous numbers of components.

Logic models offer a conceptually simple representation of biology that is easy to simulate. They are naturally suited to exploring large-scale biological networks where causality links are being hypothesized, or sought: genome, transcriptome, proteome, metabolome, interactome, microbiome - the list goes on. We are witnessing an unprecedented increase in the amount and quality of data available for describing and modeling biology at the cellular level. Graphical representation of these data as a network of (putative) relationships with nodes and edges (Figure 1d), in its many variants, is now so common that it can be considered iconic [1]. As the -ome names imply, we expect such data to be complete collections of components and/or properties. The problem is that they are neither complete nor correct. It has been argued that they often do not help understanding, and have occasionally been called the 'ridiculome'. While this is obviously tongue-in-cheek, it does reflect some real limitations. But if logic models are so easy to compute, can't they be used to test, correct, and refine large-scale models based on the existing complement of available -omics data? Actually they are, successfully so: they are the bread and butter of network inference, which aims to reverse-engineer the relationships between intracellular components responsible for regulating cellular function. In most cases we still do not know many of the interactions between various gene products, signaling molecules, metabolites, and so on, and how they lead to a particular cellular phenotype. Phenotypes characterized by high-throughput experimental measurements of state parameters (protein or mRNA expression levels, enzymatic activities, metabolite levels, and so on) can then be used to 'train' logic models that eventually will infer the putative network responsible for the observed behavior [2] (thus the 'network inference' designation).

## Peeking under the rug

**Figure 2:**
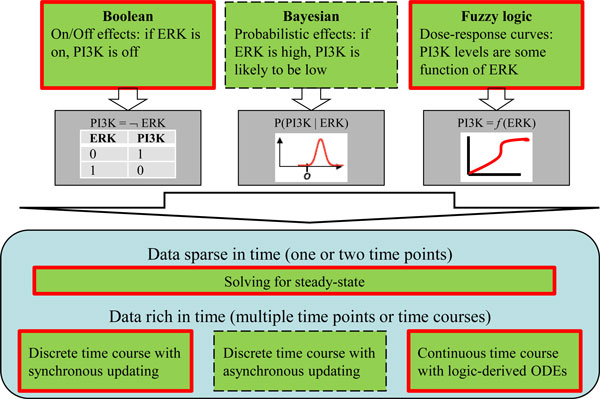
**Logic models**. Models can be encoded using simple Boolean logic (nodes may accept only true or false value), Bayesian probability (node values represent likelihood of events), or fuzzy logic (nodes have 'variable degrees of truth'). Depending on the availability of time-resolved data, these can be simulated to describe the system at steady state (one or a few selected time points), or dynamically (time course as a discrete sequence or as a continuous function). Solid red lines show methods implemented by the CellNOptR toolkit. ODE, ordinary differential equation.

The fact that logic models are easy to compute makes them useful for random searches and screening (for example, analyzing perturbations at multiple elements of the network), and for processing large amounts of individualized data (for example, comparing proteomics data from tumor cells or mutants with data from their normal counterpart). They therefore generated a lot of excitement because they appear very attractive for fields such as drug discovery [3]. But how accurate and useful are they? Some may argue that the practical results have been somewhat disappointing, but we believe this is mostly a misperception created by too high expectations, too early. A major problem is that the simple causal links that are being depicted hide an underlying complexity that is often essential to explain real world functionality: so much is swept under the rug. Of course, systems modelers are well aware of that, and cell biology has forced them to go well beyond simple Boolean logic. As a result, several more complicated logic-derived modeling approaches have been developed [4] (Figure 2). Models can be refined by replacing the simple on/off logic with probabilistic functions, such as in Bayesian networks. These can account for more graded responses, and for the stochastic effects of noise and of small numbers of molecules, as well as better represent uncertainty in the model. Even more fine-grained relationships can be encoded by using continuous transfer functions (such as linear, polynomial, or Hill-type functions) - so-called fuzzy logic. Arguably, any level of detail could be achieved by simply increasing the number of elementary links in a network and adjusting the mathematical functional form assigned to them.

Logic-derived models differ not only in the level of fine-graining of the functional relationships, but also in their ability to handle time - the dynamics of the systems. Boolean networks were originally designed to provide simple input-output relationships - that is, the steady-state achieved under varying conditions. This is appropriate, for example, for analyzing traditional transcriptomics or proteomics experiments. Whether we measure expression levels before or after some external perturbation (for example, applying a stimulus or drug), or compare different cell populations, it is still just a collection of different steady-states. True time-course data were typically limited to small scale experiments, but are now becoming available also in high-throughput technologies. Algorithms to allow logic-derived models to simulate dynamic systems aim to retain the simplicity of Boolean networks but with a fine-grained representation of time (Figure 2). Time discretization with synchronous updating is the simplest approach, where we can think of the system simply stepping through time from steady-state to steady-state. At the other end of the spectrum is continuous time representation: algorithms were recently developed to infer logic-based differential equations [5], and then simulations become similar to those of traditional kinetic models.

More detail comes with the burden of increasing computational complexity and the risk of over-parameterizing: the extensions to logic models described above require both choosing a functional form and inclusion of additional parameters such as coefficients and thresholds, all of which are often arbitrary or at best phenomenological. Von Neumann once famously quipped that 'With four parameters I can fit an elephant, and with five I can make him wiggle his trunk' (in fact, this was recently rigorously proven to be true [6]). High throughput experiments nowadays generate large amounts of data at such a rapid pace that we have trouble making sense of it all, but modelers complain that they still lack the data required to build sensible large-scale quantitative models. Having enough data to constrain the model is critical to avoid simulating phantoms.

## Is modeling software only for the initiated?

The right choice of mathematical formalism thus depends on both the purpose of the model and the type, quantity, and quality of data at hand - and for systems of any complexity will most likely be a combination of multiple methods. This was (perhaps painfully) reinforced recently by the report by Karr *et al. *[7] of the first arguably successful comprehensive model of one of the simplest existing prokaryote species. This required a huge effort of software assembly, using many different modeling approaches and countless hours of manual data mining. The overall model is described in a 100+ page supplement, has thousands of parameters, and was implemented by using custom code development as well as 20 third-party software tools. It certainly looks daunting to reproduce and expand upon this work. If we look more closely, though, we will find that in addition to the superb publication materials, there is extensive information on several project-related websites, ranging from interactive browsing of the assembled knowledgebase for the model, to fully packaged downloadable code that is 'ready to run' (that is, if you have access to compute clusters, Matlab, and more, and the expertise to configure it all). Given adequate computer resources, re-enacting the published simulations may not take more than a stubborn graduate student's few sleepless nights. But to be able to build something similar in the context of your own data from other cell types, that is a different story altogether.

The gaps and uncertainties in the knowledge of networks are still prevalent in most cases, but custom -omics data are now much easier to obtain. So perhaps advances in logic-based modeling could help. A recent paper in *BMC Systems Biology *[8] presents an integrated platform for logic-derived modeling (CellNOptR) that enables users to navigate seamlessly between many of the different formalisms discussed above, allowing for different levels of detail in both time and state, and also providing the ability to combine network inference (prediction of the network topology from experimental data) with existing curated pathway information. This work could also appear intimidating for the non-expert. CellNOptR stands for Cellular Network Optimizer R, and is implemented as a Bioconductor [9] package. One might ask whether we need to be familiar with R (a statistical programming language) and/or Bioconductor (a public collection of software tools that use R, focused mostly on manipulation and analysis of genomic-related data) in order to use CellNOptR. Maybe, maybe not. But you do not need to be a programmer to use highly sophisticated computer software, just like you do not need to be an optical engineer to use a highly sophisticated confocal microscope. Bioinformatics-savvy users may prefer to invoke CellNOptR from their own R scripts, but those unfamiliar with such programming can simply stay within the cosy confines of Cytoscape [10] (a graph/network visualization software that is very popular among biologists) where they can install a simple plugin (CytoCopteR, distributed with the CellNOptR package) that provides all functionality in a user-friendly graphical interface form. Of course, we would expect a learning curve for new users. Microscope or software, one needs to learn how to use it, and, perhaps more importantly, to learn what one can expect to accomplish by using it in terms of both capabilities and limitations.

**Figure 3:**
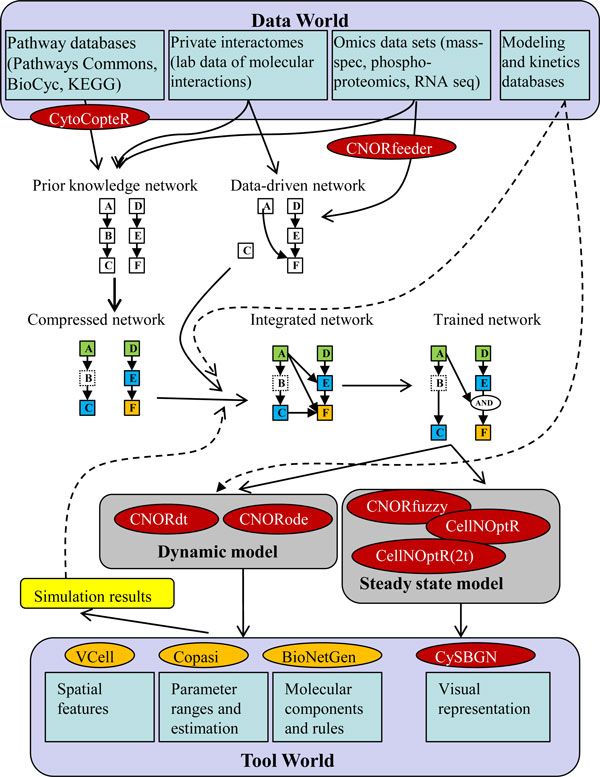
**Data to model pipeline**. The data world (public and private pathway databases/interactomes and -omics data sets supplementing existing models and kinetics data) is used to inform the model building process via several processes: building of a prior knowledge network, of a data-derived (inferred) network, and their refinement and training to data (color coding and terminology are described in [8]). Red ovals represent components of the CellNOptR toolkit and orange ovals represent other representative computational tools outside the logic model formalism. Simulation of models generated from existing data sets can be performed via different methods, not limited to ordinary differential equation or logic models. Data not used initially (such as spatial features, information about molecular domains, or wider parameter range), and the new simulation results can be used iteratively to refine model building process. Potential connections between different modeling and simulation approaches (currently only possible via ad-hoc implementations) are shown as dashed lines.

A collection of logic-derived model simulators implementing several algorithms in a single free open-source package would normally be regarded as an incremental advance. But here the whole is much larger than the sum of its parts. As mentioned before, the ease of simulating logical models makes them adept for the difficult task of reverse engineering. Indeed, CellNOptR was designed primarily to be used as a network inference tool, but using a novel approach (Figure 3), that goes beyond the usual attempt to reconstruct causal links from a particular experimental dataset with little or no prior knowledge of the system. In other words, traditional inference methods create a purely data-derived network and do not take advantage of existing knowledge about molecular interactions from different sources. Nowadays, however, such prior knowledge is often abundant. Public pathway databases store information about an enormous number of entities usable for modeling, and about the interactions among them. To wit, Pathway Commons [11], an aggregator of biological databases, currently stores more than 442,000 interactions among more than 86,000 physical entities involved in 1,668 pathways across 414 organisms; the BioCyc collection of pathway/genome databases [12] describes the genome and metabolic pathways of 1,962 distinct organisms; and there are other such collections. CellNOptR makes use of such information, and has been named a network optimizer for a reason. It takes two required inputs: first, components/interactions from a 'known' biological network containing the molecules/complexes of interest (the prior knowledge network - a.k.a. PKN), and second, an actual (usually experimental) dataset; the PKN will then be adjusted such that a simulated logic-derived model will best match the data at hand. A lot of information has been already assembled and curated into what we call canonical prior knowledge (well characterized fully referenced data stored in electronic exchange formats such as BioPAX [13]), which can be used as a starting point for models. The sheer scope of such information makes the use of it in its entirety as a PKN very difficult. Serious pre-processing by specialized tools (such as the BiNoM [14] or CytoCopteR plugins for Cytoscape) is needed, and ideally these have to be integrated with both databases and modeling tools.

## The holy grail of cellular models

Why is this a powerful approach? Because it can greatly help to understand the system being modeled. We do not wish to engage here in discussing the meaning of 'understanding' and of the usefulness of models; these have been frequent topics in biological discourse in recent years. We will rather illustrate by a hypothetical example, in very broad and practical terms (the interested reader is referred to [8] and references 11, 14, 19, 35, 40, 44 cited therein for detailed descriptions of real world examples and algorithm testing/validation). Suppose we want to investigate how signaling and gene regulation via the epidermal growth factor and tumor necrosis factor-α receptors may be altered in human hepatocarcinoma cells. We would select information available in pathway databases and a relevant transcriptomics and/or proteomics experimental dataset (typically readouts after various perturbations), and then put CellNOptR to work. Some likely results of this exercise in logic modeling could be the predictions that, in contrast to other cells, in these cancer cells the tumor necrosis factor-α receptor does not activate phosphoinositide 3-kinase, both Map3K1 and Map3K7 are required to activate MKK4, an inhibitory link from ERK to SOS-1 may be present, and so on. This context-specific model refinement provides concrete hypotheses: maybe a putative interaction shown in a yeast two-hybrid experiment does not occur *in vivo*, or maybe the transformed cell line phenotype is simply different from the canonical pathway. The latter may prove to be critical information for identifying the mechanisms that cause the hepatocarcinoma cells to respond to stimuli differently than their normal liver cell counterparts. If the experimental data have detailed time-course readouts, the differences obtained when fitting via the different algorithms could lead to additional conclusions, such as the Ras activation of Map3K1 exhibits hypersensitivity, whereas the branch linking Map3K7 to NFκB inhibition is linear and robust to perturbations - perhaps critical information for identifying potential drug targets. The mechanistic insights and predictive power are much higher than what can be obtained from purely data-driven models or simulating purely pathway-derived models.

How does this relate to large multi-scale models? Covert and colleagues [7] were able to develop a whole-cell model of the bacterium *Mycoplasma genitalium *that accounts for all molecular components and their interactions, from electrolytes and metabolites to proteins and ribosome assemblies. This highly complex model was constructed by coordinating sub-models for each of 28 classes of cellular processes, a majority of which were mathematically represented by logic-derived models of some sort (see chapter 3 of supplement S1 in [7] for details). The software and methods developed by Saez-Rodrigues and colleagues [8] make a strong statement about the power of sophisticated logic-derived models for systems such as mammalian cells, where large parts of the molecular networks are not well understood, incomplete and with unknown parameters. But the bottom panel in Figure 2 of [8] provides for both a reality-check of current capabilities and a hint of things to come. Certain behaviors of the studied system (for example, the NFκB oscillations) can be captured only by using the logic-derived differential equations (a method that is considerably more computationally expensive, and which involves many additional arbitrary parameters). This may come as no surprise. Practitioners of detailed, quantitative, validated models have preached for a long time the importance of non-linear dynamics of intracellular molecular interactions, especially in signaling networks, but often also in metabolic or gene regulatory networks (in fact, these classifications of networks are increasingly blurred nowadays). Detailed studies have shown that parts of these networks can act as modules with distinct dynamical features (threshold, hysteresis, oscillatory instability, switch-like instability, and so on) [15]. Such emergent properties may be due not only to network topology, but to the detailed kinetic rate laws and quantitative parameters. To complicate matters further, impedance effects sometimes change individual module behavior when multiple modules are connected to each other.

In fact, much more is swept under the rug than we have alluded to so far. Even the simple cartoon diagram shown in Figure 1a embodies more information than the simple causal links captured by the logic models shown in Figure 1b. Multiple phosphosites can create a combinatorial complexity of regulatory actions, and difficulties in mapping functional states to measured observable quantities. Compartments, scaffolds, and diffusion create spatial inhomogeneities and microdomains, which have critical functionality in many eukaryotic cells (often also in prokaryotes) [16]. And to top it all off, there has been increasing evidence that parts of the cellular machinery employ fleeting, non-stoichiometric, pleiomorphic assemblies of molecules to carry out vital processes [17]. Many novel methods and algorithms have been developed in recent years by the 'bottom-up' modelers and experimentalists to tackle these problems: rule-based [18] and network-free models [19], spatially resolved models with continuous representations (partial differential equations-based) [20] or with discrete representations (particle-based stochastics) [21], as well as refinement of methods long used in mathematical biology, such as agent-based simulation methods and constraint-based models. New theoretical methods and software applications continuously appear in different areas related to modeling, ranging from network-based approaches for predicting missing pathway interactions [22] to multi-level rule-based modeling [23].

Does this detract from our praise of the advances in logic-derived models discussed above? No. To the contrary, this is why we are really excited. Let us return to those logic-derived ordinary differential equations (ODEs and how they (can) relate to the other side of the field. Of course, they are phenomenological constructs of whatever arbitrary mathematical form is being provided (in this case Hill-type equations, which can capture a variety of common non-linear relationships with only two parameters). But such mathematical approximations are sometimes the starting point for discovering the underlying mechanism. In what is arguably one of the most influential modeling works related to biology, almost exactly 100 years ago Leonor Michaelis and Maud Menten used a phenomenological equation to fit the experimental measurements of the initial velocity of the invertase-catalyzed reaction (at time zero, when no product has formed yet, the reaction can be simplified and modeled as being irreversible). Based on that approximation, they posited that the enzyme activity could be explained by mass-action kinetics involving an intermediary reaction complex - the fundamental mechanism of enzymatic catalysis that was confirmed three decades later [24]. The fact that explaining certain qualitative characteristics requires the ODE-based formalism is the perfect starting point for directing new detailed investigations of potential mechanistic hypotheses.

Moreover, if we can modify a logic-derived model and end up with a differential equations-based model, why not jump over the fence and use what is available in the world of kinetic models? For starters, much more powerful optimization algorithms and tools have been developed in that domain [25]. Taking advantage of these would be trivial if models could be exported into a community standard format such as Systems Biology Markup Language (SBML) [26]. And if support for this exchange format were implemented in reverse, too, one could import pre-existing detailed kinetic models into software that deals with logic formalism as just another form of prior knowledge for those interactions where such information already exists (for reference, as of this writing, the Biomodels database makes available 154,456 kinetic relationships between 133,559 molecular species in the curated branch).

It sounds trite to say that we need to use multiple approaches and tools in order to build truly complete and accurate cellular models. We are getting closer not only to integrating multiple logic-based formalisms easily, but also to crossing over into kinetic, spatial, rule-based models, and more. And the experimental data required for building all these different types of computational models at different scales and levels of detail will have to come from both 'small science' and 'big science' [27].
